# Short-term outcomes after transplantation of deceased donor kidneys with acute kidney injury: a retrospective analysis of a multicenter cohort of marginal donor kidneys with post-explantation biopsies

**DOI:** 10.1007/s11255-022-03277-3

**Published:** 2022-07-09

**Authors:** Florian G. Scurt, Angela Ernst, Tamara Wassermann, Ben Hammoud, Peter R. Mertens, Anke Schwarz, Jan U. Becker, Christos Chatzikyrkou

**Affiliations:** 1 University Clinic for Nephrology and Hypertension, Diabetology and Endocrinology, Medical Faculty, Otto-Von Guericke University Magdeburg, Magdeburg, Germany; 2 Institute of Medical Statistics and Bioinformatics, University of Cologne, Cologne, Germany; 3 Department of Nephrology and Hypertension, Hannover Medical School, Hannover, Germany; 4 Institute of Pathology, University Hospital of Cologne, Cologne, Germany

**Keywords:** Acute kidney injury, Transplantation, End-stage kidney disease, Delayed graft function

## Abstract

**Background:**

Deceased donor kidneys with acute kidney injury (AKI) are often discarded because of concerns about inferior transplant outcomes. A means of grading the quality of such kidneys is the performance of procurement biopsies.

**Methods:**

This is a retrospective study of 221 brain death donors with marginal kidneys transplanted in 223 recipients in Germany. Marginal kidneys were defined as kidneys with procurement biopsies done exceptionally to assess suitability for transplantation in otherwise potentially discarded organs. The impact of deceased donor AKI on patient survival and death-censored graft survival at 1, 3 and 5 years and graft function at 1 and 3 years after transplantation was investigated.

**Results:**

Recipients of kidneys with stage 3 AKI had a greater incidence of delayed graft function [DGF; OR_Stage 1_: 1.435 (95% CI 0.438–0.702), OR_Stage 2_: 2.463 (95% CI 0.656–9.245), OR_Stage 3_: 4.784 (95% CI 1.421–16.101)] but a similar graft and patient survival compared to recipients of donors without AKI and with AKI stage 1 and 2 as well. The coexistence of recipient DGF and donor AKI was associated with the lowest graft survival and function rates.

**Conclusion:**

The transplantation of deceased donor marginal kidneys with AKI confers a higher risk for DGF but is associated with acceptable graft and patient outcomes, which do not differ in comparison with marginal donor kidneys without AKI. Graft prognosis is especially poor if donor AKI and recipient DGF concur. Donor AKI was a risk factor independent of the histological lesions of procurement biopsies.

**Supplementary Information:**

The online version contains supplementary material available at 10.1007/s11255-022-03277-3.

## Introduction

Kidney transplantation (KT) continues to remain the best available renal replacement therapy for most patients with end-stage renal disease. The shortage of organs and the continuously increasing number of patients on the waiting list led to the increased usage of organs from marginal donors [1]. The challenge with marginal kidneys is that delayed graft function (DGF) occurs frequently and may be associated with inferior graft and patient outcomes [2, 3].

Acute kidney injury (AKI) is very common in kidney donors and is strongly correlated with DGF [4–8]. Previous studies reported that the prognosis of KT from donors with AKI does not significantly differ from that of KT from donors without AKI. In contrast, other studies indicated that AKI does have an impact on long-term allograft outcome [7, 9]. Thus, the impact of donor AKI on allograft outcome is unknown and donor kidneys are discarded at a higher rate [6, 10]. Unfortunately, most studies focused on the analysis of KT performed with standard criteria donors. Therefore, it is unclear whether kidneys with AKI recovered from expanded criteria donors (ECDs) or from donors with a high kidney donor profile index (KDPI) or from donors with marginal organ quality exhibit similar results. Another important point is whether procurement biopsies are helpful in such cases [10].

Given these open questions, we aimed to investigate AKI in kidneys from marginal donors with post-explantation biopsies. We evaluated the impact of clinical donor characteristics and histological findings of their biopsies on short-term patient and graft survival and short-term graft function.

## Materials and methods

### Study population

We extracted data from the Deutsche Stiftung Organtransplantation (DSO) Region Nord and from the German transplant centers of kidneys allocated between January 2003 and March 2012. We included adult recipients of deceased-donor kidney-only transplants of marginal organ quality in Germany. Recipients were excluded if they were < 18 years old at the time of transplantation, if they received multiple types of organs, or if their donors were from outside of Germany. According to German regulations, only brain-dead donors were included in the study. For the same reason, normothermic ex vivo kidney perfusion systems for organ preservation were also not used.

Donor variables, procurement biopsy results, recipient variables and transplant factors included in the analysis are listed in Tables 1, 2 and 3.

**Table 1:** Baseline characteristics of donors without and with AKI

	Non-AKI (*n* = 134)	AKI (*n* = 89)	*P* value
Donor characteristics			
Age (y)	64.5 ± 15.4	56.9 ± 17.6	**0.001**
Sex [*n* (%)]			
Female	63 (53.0)	45 (50.6)	0.723
Male	71 (47.0)	44 (49.4)	
BMI (kg/m^2^)	25.8 ± 4.4	28.7 ± 6.6	** < 0.001**
Diabetes mellitus [*n* (%)]	10 (7.5)	15 (16.9)	**0.029**
Hypertension [*n* (%)]	76 (56.7)	45 (50.6)	0.366
Cardiovascular disease [*n* (%)]	38 (28.6)	22 (24.7)	0.526
Smoker [*n* (%)]	29 (21.6)	24 (27.0)	0.360
Hepatitis B Virus positive [*n* (%)]	12 (9.0)	3 (3.4)	0.103
Hepatitis C Virus positive [*n* (%)]	1 (0.7)	1 (1.1)	0.770
Cytomegalovirus positive [*n* (%)]	89 (66.4)	64 (71.9)	0.387
Traumatic brain injury	32 (23.9)	2 (2.2)	** < 0.001**
Cerebrovascular accident (CVA) [*n* (%)]	76 (56.7)	61 (68.5)	0.076
Expanded criteria donors [*n* (%)]	101 (75.4)	52 (58.4)	**0.008**
Kidney donor risk index (KDRI)	1.552790997 ± 0.506012133	1.425651457 ± 0.516209793	0.050
Kidney donor profile index (KDPI) groups [*n* (%)]			
Group 1: 0–20%	8 (6.0)	5 (5.6)	0.086
Group 2: 21–40%	8 (6.0)	9 (10.1)	
Group 3: 41–60%	14 (10.4)	20 (22.5)	
Group 4: 61–80%	23 (17.2)	12 (13.5)	
Group 5: 81–100%	81 (60.4)	43 (48.3)	
Donor ICU data			
Time ICU until confirmed brain death (h)	107.2 ± 136.5	152.9 ± 119.3	** < 0.001**
Time ICU until cross-clamp (h)	119.7 ± 138.3	158.9 ± 119.9	** < 0.001**
Duration of brain death (h)	14.9 ± 16.6	13.0 ± 16.8	0.145
Time incision until cross-clamp (min)	48.9 ± 28.0	56.6 ± 32.6	0.071
Time cross-clamp until ectomy right kidney (min)	42.6 ± 14.3	46.2 ± 20.8	0.648
Time cross-clamp until ectomy left kidney (min)	48.9 ± 14.8	51.3 ± 23.1	0.807
CPR at ICU stay [*n* (%)]	25 (18.7)	18 (20.2)	0.771
Transfusion at ICU stay [*n* (%)]	12 (9.0)	14 (15.7)	0.123
Volume expander at ICU stay [*n* (%)]	29 (21.6)	8 (9.0)	**0.013**
Diuretics at ICU stay [*n* (%)]	11 (8.3)	15 (16.9)	0.054
Antidiuretics at ICU stay [*n* (%)]	45 (34.1)	30 (33.7)	0.953
Steroids at ICU stay [*n* (%)]	35 (26.5)	12 (13.5)	**0.020**
Antibiotics at ICU stay [*n* (%)]	64 (47.8)	48 (53.9)	0.367
Serum creatinine (µmol/l)			
Admission	95.9 ± 46.1	141.2 ± 109.6	** < 0.001**
Peak	103.9 ± 50.0	290.3 ± 177.6	** < 0.001**
Terminal	99.8 ± 49.5	263.4 ± 181.8	** < 0.001**
eGFR, ml/min/1.73 m^2^ (CKD-EPI)			
Admission	71.6 ± 26.2	57.0 ± 27.4	** < 0.001**
Peak	66.4 ± 26.0	25.6 ± 16.9	** < 0.001**
Terminal	69.3 ± 26.6	32.3 ± 23.8	** < 0.001**
RIFLE criteria [*n* (%)]			
No AKI	134 (100.0)	–	–
Risk	–	32 (36.0)	
Injury	–	21 (23.6)	
Failure	–	36 (40.4)	
Urine volume last 24 h before cross-clamp (ml)	3657 ± 2478	2987 ± 2322	**0.032**
Urine volume last 24 h before cross-clamp (ml/kg)	48.2 ± 36.9	35.2 ± 29.0	**0.002**
Urine volume last hour before cross-clamp (ml)	283.6 ± 768.1	129.3 ± 152.4	** < 0.001**
Urine volume last hour before cross-clamp (ml/kg)	3.6 ± 9.1	1.6 ± 1.8	** < 0.001**
Urine test strip before cross-clamp (%) (negative/slightly positive/strong positive)			
Protein	70.1/25.4/4.5	51.7/37.1/11.2	**0.013**
Leukocytes	70.2/20.2/9.5	67.3/19.2/13.5	0.776
Red blood cells	52.4/40.2/7.3	43.1/45.1/11.8	0.491

Numbers in bold are statistically significant (for those with a *P* < 0.05)

Continuous variables are presented as mean ± standard deviation

*AKI*, acute kidney injury, *BMI* body mass index, *CPR* cardiopulmonary resuscitation, *DGF* delayed graft function, *dl* deciliter, *g* gram, *h* hours, *IU* international units, *kg* kilogram, *l* liter, *ml* milliliter, *min* minutes, *mmHg* millimeter of mercury, *mmol* millimole, *m*^*2*^ square meter, *s* seconds, *y* years, *µg* microgram

**Table 2:** Macroscopic and microscopic characteristics of donor kidneys with and without AKI

	Non-AKI (*n* = 134)	AKI (*n* = 89)	*P* value
Macroscopic characteristics			
Perfusion’s quality, % (good/medium/bad)	93.3/3.7/3.0	96.6/2.2/1.1	0.531
Organ quality, % (good/medium/bad)	74.6/22.4/3.0	73.0/27.0/0.0	0.208
Histopathological characteristics			
Glomerulosclerosis (%)	11.3 ± 15.5	9.0 ± 14.3	**0.019**
Glomerulosclerosis > 5 (%)	58.4	37.1	**0.006**
Banff lesion scores (%)			
Interstitial inflammation (i) ≥ 1	13.9	18.6	0.406
Tubulitis (t) ≥ 1	12.9	8.6	0.379
Intimal arteritis (v) ≥ 1	1.0	0.0	0.404
Glomerulitis (g) ≥ 1	16.8	10.0	0.206
Peritubular capillaritis (ptc) ≥ 1	0.0	0.0	> 0.999
Interstitial fibrosis (ci) ≥ 1	24.8	12.9	0.055
Tubular atrophy (ct) ≥ 1	46.5	27.1	**0.016**
Vascular fibrous intimal thickening (cv) ≥ 1	70.3	41.4	** < 0.001**
GBM double contours (cg) ≥ 1	2.0	4.3	0.379
Mesangial matrix expansion (mm) ≥ 1	24.8	8.6	**0.007**
Arteriolar hyalinosis (ah) ≥ 1	72.3	54.3	**0.015**
Interstitial fibrosis and tubular atrophy (IFTA) ≥ 1	35.8	20.2	**0.012**
Arteriolar fibrosis (0/1/2/3) (%)	40.6/47.5/10.9/1.0	74.3/17.1/7.1/1.4	** < 0.001**
Thrombotic microangiopathy (%)	5.9	7.1	0.753
Diabetic nephropathy (%)	7.7	2.0	0.174
Nephrocalcinosis % (Nein/Gering/Mäßig/Schwer)	90.0/5.0/5.0	87.1/4.3/8.6	0.631
Tubular hypertrophy (%)	19.8	18.6	0.841
Epithelial cell flattening (0/1/2/3) (%)	2.0/48.5/24.8/24.8	5.7/28.6/44.3/21.4	**0.013**
Brush border membrane defect (0/1/2/3) (%)	1.0/31.7/48.5/18.8	1.4/20.0/44.3/34.3	0.100
Vacuolization (0/1/2/3) (%)	6.9/28.7/22.8/41.6	7.1/14.3/21.4/57.1	0.117
Loss of nuclear staining (0/1/2/3) (%)	1.0/32.7/35.6/30.7	2.9/20.0/41.4/35.7	0.270
Cellular detritus (0/1/2/3) (%)	12.9/39.6/28.7/18.8	20.0/42.9/15.7/21.4	0.206
Pyelonephritis positive (%)	7.9	8.6	0.879

Numbers in bold are statistically significant (for those with a *P* < 0.05)

Continuous variables are presented as mean ± standard deviation

**Table 3:** Baseline characteristics and transplantation data of transplanted patients receiving donor kidneys with and without AKI

	Non-AKI (*n* = 134)	AKI (*n* = 89)	*P* value
Recipients’ characteristics			
Age (y)	61.0 ± 13.0	61.0 ± 14.2	0.989
Sex [*n* (%)]			
Female	48 (64.2)	27 (69.7)	0.396
Male	86 (35.8)	62 (30.3)	
BMI (kg/m^2^)	25.6 ± 4.3	25.4 ± 4.5	0.675
Diabetes mellitus [*n* (%)]	34 (25.4)	25 (28.1)	0.652
Hypertension [*n* (%)]	117 (87.3)	74 (83.1)	0.385
Cardiovascular disease [*n* (%)]	59 (44.4)	36 (39.4)	0.887
HBsAg positive [*n* (%)]	34 (25.6)	15 (16.9)	0.125
Hepatitis C Virus positive [*n* (%)]	0 (0.0)	6 (6.7)	**0.002**
Cytomegalovirus positive [*n* (%)]	92 (68.7)	56 (62.9)	0.375
Pretransplant dialysis interval (months)	169.5 ± 79.4	163.1 ± 79.2	0.559
Prior organ transplant [*n* (%)]	15 (11.2)	9 (10.1)	0.799
Raw estimated post-transplant survival (EPTS)	2.676527162 ± 0.609486574	2.648425146 ± 0.644190489	0.742
Estimated post-transplant survival (EPTS) groups [*n* (%)]			
Group 1: 0–20%	11 (8.3)	7 (7.9)	0.471
Group 2: 21–40%	7 (5.3)	7 (7.9)	
Group 3: 41–60%	19 (14.3)	10 (11.2)	
Group 4: 61–80%	14 (10.5)	16 (18.0)	
Group 5: 81–100%	82 (61.7)	49 (55.1)	
Transplant baseline characteristics			
HLA-A mismatch (0/1/2) (%)	15.7/50.7/33.6	12.4/50.7/33.6	0.142
HLA-B mismatch (0/1/2) (%)	7.5/46.3/46.3	9.0/52.8/38.2	0.489
HLA-DR mismatch (0/1/2) (%)	13.4/52.2/34.3	15.7/59.6/24.7	0.311
Negative PRA at transplantation [*n* (%)]	118 (88.1)	82 (92.1)	0.327
Average PRA at transplantation	3.0 ± 11.3	1.4 ± 6.5	0.216
Historic peak of PRA	8.0 ± 21.8	5.4 ± 17.4	0.684
Origin of donor kidney (r/l/b) (%)	49.6/48.7/1.8	51.8/47.3/0.9	0.824
Cold ischemia time (h)	13.9 ± 5.2	13.7 ± 4.8	0.829
Warm ischemia time (min)	39.8 ± 14.04	41.6 ± 14.2	0.359
Maintenance therapy			
Calcineurin inhibitors, % (cyclosporin/tacrolimus/other)	76.2/22.2/1.6	73.8/26.3/0.0	0.465
Anti-metabolites, % (azathioprine/mycophenolate/other)	1.6/87.5/10.0	0.0/81.5/18.5	0.250
mTOR inhibitors (%)	1.6	6.3	0.163
Steroids (%)	92.2	90.1	0.666

Numbers in bold are statistically significant (for those with a *P* < 0.05)

Continuous variables are presented as mean ± standard deviation

*b* both, *BMI* body mass index, *h* hours, *HBsAg* HBV surface antigen, *kg* kilogram, *l* left, *m*^*2*^ square meter, *min* minutes, *PRA* panel reactive antibodies, *r* right, *y* years

### Definitions

Marginal kidneys were defined according to the current clinical practice in Germany, i.e., kidneys with procurement biopsies done exceptionally to assess suitability for transplantation in otherwise potentially discarded organs. Such kidneys had, for example, proteinuria or presumed chronic kidney disease, were of poor macroscopic or perfusion quality, had heavy aortic patch and/or renal artery atherosclerosis, had multiple accessory renal arteries or were recovered from donors with long ICU stay, diabetes and multiorgan failure. Macroscopic grading of the external aspect of the donor kidney was provided by the explanting surgical team as good, medium, or poor; likewise, atherosclerosis was characterized as no, mild or severe and perfusion quality as good, medium or poor. Extended criteria donors (ECD) were classified as brain death donors 60 years of age or donors 50 to 59 years of age with at least 2 of the following features: history of hypertension, terminal serum creatinine > 1.5 mg/dl or cerebrovascular cause of death [11].

The original Chronic Kidney Disease Epidemiology Collaboration (CKD-EPI) equation was used to calculate eGFR [12]. AKI was defined as per the Kidney Disease: Improving Global Outcomes (KDIGO) criteria [13]. DGF was defined as the need for dialysis within the first 7 days of transplantation similar to many papers focusing on this topic [14]. Duration of brain death was defined as the interval between brain death and the beginning of cold ischemia time and was calculated by subtracting the time of ICU until cross-clamp, where cold perfusion started, and the time of ICU until declaration of brain death.

Overall graft loss was defined as time from transplantation to return to dialysis, or death with a functioning graft. Death-censored graft failure was the same apart from censoring those who died with a functioning graft. Patient death was defined as the time from date of transplantation to patient death, not censored at graft failure. All survival times were censored at the end of follow-up or loss to follow-up.

### Outcome measures

Four different outcomes were analyzed: (1) primary non-function (PNF), (2) DGF, (3) recipient eGFR/creatinine at 3, 12 and 36 months, and (4) graft loss, death-censored graft failure and patient survival at 1 and 3 years.

### Histological assessment of procurement biopsy

All biopsies were processed in paraffin according to the routine protocol at the Institute of Pathology, Hannover Medical School, which involves multiple level sections stained with hematoxylin and eosin, periodic-acid Schiff, Jones silver, trichrome elastica. Histopathological parameters were retrospectively determined by an experienced nephropathologist and included type of biopsy, total number of glomeruli and ratio of globally sclerosed glomeruli, focal and segmental glomerulosclerosis (FSGS), number of arteries (media ≥ 2 smooth muscle cell layers), presence of FSGS, Banff Lesion Scores i, t, v, g, ptc, ci, ct, cv, cg, ah according to Banff 2011 [15–18], arteriolar fibrosis scored as absent, mild, moderate, severe, cortical tubular hypertrophy, epithelial cell flattening, brush border loss, vacuolization, luminal detritus as 0 (absent), 1 (< 25%), 2 (< 50%), 3 (≥ 50%), tubular nuclear loss 0 (absent), 1 (1 quadrant), 2 (2 quadrants), 3 (3 quadrants of the most affected tubular cross section), pyelonephritis, thrombotic microangiopathy as glomerular microthrombi.

### Statistical analysis

The baseline characteristics of the study cohort were expressed as mean (SD). We created logistic regression models for the outcome of DGF, adjusting for covariates. The linearity assumption was assessed through categorization of continuous variables. We checked for interaction terms using forward elimination. Nonsignificant variables were removed from the model using backward elimination with a cutoff of *P* < 0.05. Variables were also considered confounders if they changed the coefficient of the explanatory variable by > 10%. The different exposure variables were inserted into the model. We compared the models using *F* test, adjusted *R*^2^ and the Hosmer–Lemeshow goodness of fit and the *C* statistic.

Three multilinear regression models for the outcomes of recipient eGFR at 3, 12 and 36 months were created, adjusting for covariates. Collinearity of different variables was assessed using the variance inflation factor. The linearity assumption was assessed using scatter plots of residual values for each continuous variable. Effect modification was assessed for using the forward elimination method. Nonsignificant variables were removed from the model using backward elimination. The different exposure variables were then assessed in the different models. The Wald test was used to assess the significance of exposure variable plus any interaction terms. We then compared the variables of interest for the different models using the *F* test and adjusted *R*^2^.

Three separate multivariable Cox proportional hazards models were created to assess the outcomes of death-censored graft failure, and patient death. Nonlinear continuous variables were made categorical. The nonsignificant variables were removed from the model using backward elimination. Wald statistics were used to assess the significance of exposure variables. The models were assessed using the Harrell *C* statistic and Akaike Information Criterion (AIC).

The variables for which the various models were adjusted for in the multivariate analyses are summarized in Supplementary File 1.

A *P* value below of 0.05 was considered significant in all two-sided tests. Statistical analysis was performed with SPSS software, v24 (IBM Corp, Armonk, NY) and IBM SPSS Statistics Essentials for R.

### Ethical approval

The study protocol was conducted in accordance with the Declarations of Helsinki and Istanbul on organ trafficking and transplant tourism and approved by the Ethics Committee of Hannover Medical School (No. 1519-2012).

## Results

### Donors’ and recipients’ characteristics

**Fig. 1:**
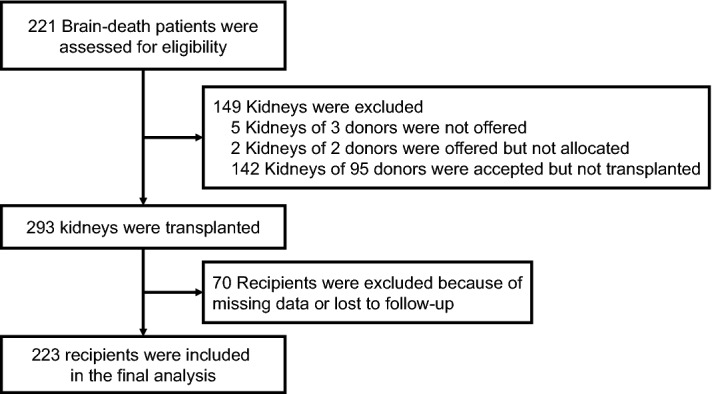
Study’s flowchart

From 442 kidneys of marginal quality considered for transplantation and with procurement biopsies, 149 were not transplanted. For the remaining 293 transplanted kidneys, follow-up data were available for 223 organs (Fig. 1).

Donors’ characteristics are summarized in Table 1. The mean age of non-AKI and AKI donors was 64.5 ± 15.6 and 56.9 ± 12.9 years (*P* = 0.001). Furthermore, the KDRI was 1.553 ± 0.506 and 1.426 ± 0.516 in the non-AKI and AKI donor groups. The mean donor BMI and the prevalence of diabetes were higher, whereas less numbers of ECDs were observed in the AKI group. No differences in donors’ gender, hypertension, smoking rate, Hepatitis B and C and CMV serology, causes of brain death and distribution of KDPI were observed between the non-AKI and AKI donor groups. Traumatic brain injury was more common in donors without AKI, but duration of brain death was comparable between groups. Donors with AKI remained for a longer time in the ICU and received less often volume expanders and steroids.

In the AKI donor group serum creatinine at admission, peak serum creatinine and creatinine and recovery were higher. Furthermore, AKI donors had more proteinuria and reduced diuresis in the last 24 h before cross-clamp.

Regarding perfusion and organ quality, there were no differences between kidneys with and without AKI (Table 2). However, post-explantation biopsies revealed more severe chronic glomerular and tubulointerstitial damage in the non-AKI group.

Table 3 presents recipients’ baseline characteristics. Of 223 recipients, 89 received kidneys from 50 AKI donors, whereas the remaining 134 received kidneys from 83 donors without AKI. A total of 153 patients (68.6%) received kidneys from ECDs. Non-immunological and immunological risk factors for renal allograft failure, such as age, history of hypertension or cardiovascular disease, prior transplantation, human leukocyte antigen (HLA) mismatches, plasma reactive antibody (PRA) titers and cold and warm ischemia time and immunosuppressive therapy did not differ between groups. Hepatitis C was more prevalent in recipients of donor kidneys with AKI.

### Analysis of clinical outcomes

**Fig. 2:**
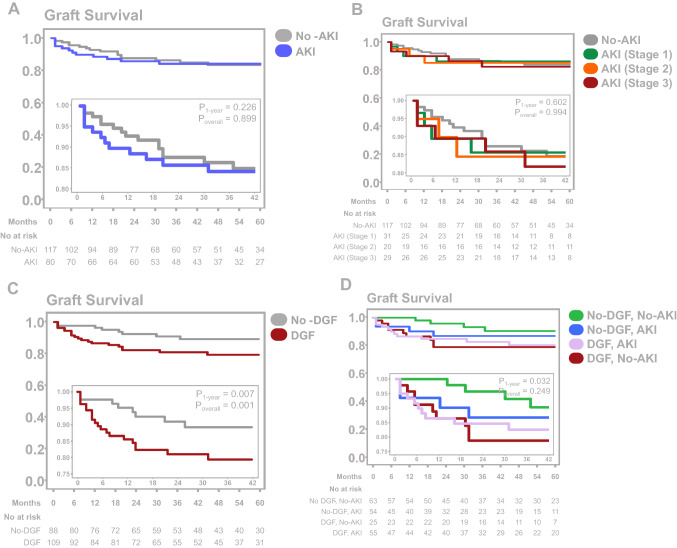
**A** Kaplan–Meier curve illustrating graft survival in AKI versus No-AKI kidney transplant recipients. **B** Kaplan–Meier curve illustrating graft survival according to AKI stages. **C** Kaplan–Meier curve illustrating graft survival in recipients with and without AKI. **D** Kaplan–Meier curve illustrating graft survival in recipients with and without DGF receiving kidneys from donors with and without AKI

Clinical outcomes are presented in Tables 4 and 5 and illustrated in Fig. 2. There were no differences in PNF of the graft between recipients of donors with and without AKI. Although DGF was more common in recipients of donor kidneys with AKI (68.8% versus 46.2%; *P* = 0.002), patient and death-censored graft survival at 1, 3 and 5 years graft function at 3 months, 1 and 3 years, proteinuria and number of rejections were similar in both groups.

**Table 4:** Rate of PNF from donors with and without AKI

	Non-AKI (*n* = 134)	AKI (*n* = 89)	*P* value
PNF [*n* (%)]	17 (12.7)	9 (10.1)	0.557

**Table 5:** Short-term outcome from donors with and without AKI

	Non-AKI (*n* = 117)	AKI (*n* = 80)	*P* value
Delayed graft function [*n* (%)]	54 (46.2)	55 (68.8)	**0.002**
Patient survival [*n* (%)]			
At 1 year	104 (88.9)	75 (93.8)	0.245
At 3 years	100 (85.5)	70 (87.5)	0.684
At 5 years	94 (80.3)	69 (86.3)	0.281
Graft survival (death-censored) [*n* (%)]			
At 1 year	97 (93.3)	66 (88.0)	0.223
At 3 years	88 (88.0)	59 (84.3)	0.486
At 5 years	77 (76.2)	62 (79.5)	0.605
Graft function (creatinine) (µmol/l)			
At 3 months	189.6 ± 85.9	186.5 ± 82.0	0.784
At 1 year	166.2 ± 62.3 (*n* = 88)	173.8 ± 51.7 (*n* = 60)	0.347
At 3 years	164.9 ± 55.0 (*n* = 48)	170.2 ± 67.7 (*n* = 33)	0.694
Graft function (creatinine) (ml/min/1.73 m^2^)			
At 3 months	36.1 ± 16.7	35.5 ± 15.2	0.304
At 1 year	40.0 ± 16.0 (*n* = 88)	37.4 ± 12.9 (*n* = 74)	0.542
At 3 years	40.6 ± 17.7 (*n* = 48)	40.0 ± 17.0 (*n* = 43)	0.917
Proteinuria at 3 months (g/day)			
At 3 months	0.34 ± 1.28 (*n* = 59)	0.26 ± 0.23 (*n* = 38)	0.706
At 1 year	0.29 ± 0.62 (*n* = 47)	0.26 ± 0.25 (*n* = 36)	0.774
Number of rejections	0.68 ± 1.14 (*n* = 70)	0.62 ± 0.94 (*n* = 46)	0.659

Numbers in bold are statistically significant (for those with a *P* < 0.05)

*AKI* acute kidney injury, *DGF* delayed graft function, *g* gram, *l* liter, *min* minute, *ml* milliliter, *mmol* millimole, *m*^*2*^ square meter

Increasing stage of donor AKI was associated with a higher rate of DGF (stage 1 in 46.2%, stage 2 in 65.0%, stage 3 in 79.3%, Tables 6, 7), but only stage 3 remained significant after multivariable logistic regression [stage 1 odds ratio (OR) 1.435 95% CI 0.438–4.702, stage 2 OR 2.463 95% CI 0.656–9.245, stage 3 OR 4.784 95% CI 1.421–16.101, Table 8, the models had moderate discrimination and were similar across all models (*C* statistics: AKI = 0.772 (CI 0.703–0.842), AKIN classification = 0.784 (CI 0.714–0.853))].

**Table 6:** Rate of PNF and DGF according to the severity of donor AKI with and without AKI

	AKIN1 (*n* = 32)	AKIN2 (*n* = 21)	AKIN3 (*n* = 36)	*P* value
PNF [*n* (%)]	1 (3.1)	1 (4.8)	7 (19.4)	0.139

**Table 7:** Short-term outcome according to the severity of donor AKI with and without

	AKIN1 (*n* = 31)	AKIN2 (*n* = 20)	AKIN3 (*n* = 29)	*P* value
Delayed graft function [*n* (%)]	19 (46.2)	13 (65.0)	23 (79.3)	**0.008**
Patient survival [*n* (%)]				
At 1 year	27 (87.5)	19 (95.2)	29 (100.0)	0.221
At 3 years	23 (74.2.)	19 (95.0)	28 (96.6)	0.051
At 5 years	23 (74.2)	18 (90.0)	28 (96.6)	0.083
Graft survival (death-censored) [*n* (%)]				
At 1 year	24 (88.9)	16 (84.2)	26 (89.7)	0.584
At 3 years	20 (87.0)	16 (84.2)	23 (82.1)	0.865
At 5 years	20 (87.0)	15 (83.3)	23 (82.1)	0.964
Graft function (creatinine) (µmol/l)				
At 3 months	191.8 ± 102.9	203.3 ± 75.7	171.9 ± 62.1	0.345
At 1 year	172.7 ± 60.2 (*n* = 23)	175.0 ± 33.5 (*n* = 14)	174.0 ± 53.6 (*n* = 23)	0.987
At 3 years	151.8 ± 56.4 (*n* = 12)	169.8 ± 48.6 (*n* = 9)	184.2 ± 83.2 (*n* = 12)	0.467
Graft function (creatinine) (ml/min/1.73 m^2^)				
At 3 months	36.7 ± 16.6	32.8 ± 14.5	40.7 ± 22.7	0.306
At 1 year	36.5 ± 13.0 (*n* = 23)	35.4 ± 11.7 (*n* = 14)	37.7 ± 15.3 (*n* = 23)	0.822
At 3 years	41.6 ± 15.0 (*n* = 12)	41.1 ± 18.2 (*n* = 9)	36.8 ± 17.1 (*n* = 12)	0.710
Proteinuria at 3 months (g/day)				
At 3 months	0.27 ± 0.26 (*n* = 14)	0.28 ± 0.32 (*n* = 8)	0.25 ± 0.16 (*n* = 16)	0.924
At 1 year	0.32 ± 0.33 (*n* = 15)	0.17 ± 0.05 (*n* = 7)	0.25 ± 0.22 (*n* = 14)	0.422
Number of rejections	0.55 ± 0.83 (*n* = 20)	0.64 ± 0.92 (*n* = 10)	0.62 ± 1.12 (*n* = 16)	0.963

Numbers in bold are statistically significant (for those with a *P* < 0.05)

*AKI* acute kidney injury, *DGF* delayed graft function, *g* gram, *l* liter, *min* minute, *ml* milliliter, *mmol* millimole, *m*^*2*^ square meter

**Table 8:** Model test statistics and values for unadjusted and adjusted AKI models for different graft and patient outcomes

	AKI (yes/no)	AKIN classification (AKIN stage 1–3)
Primary non-function		
OR (95% CI)	0.774 (0.329 to 1.823)	AKIN_Stage 1_: 0.222 (0.028 to 1.734)
		AKIN_Stage 2_: 0.344 (0.043 to 2.732)
		AKIN_Stage 3_: 1.661 (0.630 to 4.380)
OR_adjusted_ (95% CI)	0.850 (0.238 to 3.040)	AKIN_Stage 1_: 0.544 (0.054 to 5.476)
		AKIN_Stage 2_: 0.864 (0.086 to 8.677)
		AKIN_Stage 3_: 1.044 (0.220 to 4.965)
*χ*^2^ Test	2.897	6.541
*C* statistic (95% CI)	0.844 (0.759 to 0.929)	0.844 (0.758 to 0.931)
Delayed graft function		
OR (95% CI)	**2.567 (1.414 to 4.660)**	AKIN_Stage 1_: 1.847 (0.823 to 4.148)
		AKIN_Stage 2_: 2.167 (0.807 to 5.820)
		**AKIN**_**Stage 3**_**: 4.472 (1.733 to 11.356)**
OR_adjusted_ (95% CI)	**2.668 (1.152 to 6.177)**	AKIN_Stage 1_: 1.435 (0.438 to 4.702)
		AKIN_Stage 2_: 2.463 (0.656 to 9.245)
		**AKIN**_**Stage 3**_**: 4.784 (1.421 to 16.101)**
*χ*^2^ Test	41.65	44.475
*C* statistic (95% CI)	0.772 (0.703 to 0.842)	0.784 (0.714 to 0.853)
Patient survival		
1-year patient survival		
HR (95% CI)	0.645 (0.236 to 1.766)	AKIN_Stage 1_: 0.911 (0.304 to 2.731)
		AKIN_Stage 2_: 0.605 (0.080 to 4.573)
		AKIN_Stage 3_: N/A*
HR_adjusted_ (95% CI)	0.692 (0.207 to 2.317)	AKIN_Stage 1_: 1.151 (0.223 to 5.931)
		AKIN_Stage 2_: 0.551 (0.059 to 5.177)
		AKIN_Stage 3_: N/A*
Wald statistic	0.357	0.285
*P* value	0.550	0.963
3-year patient survival		
HR (95% CI)	1.014 (0.476 to 2.158)	AKIN_Stage 1_: 1.515 (0.667 to 3.438)
		AKIN_Stage 2_: 0.447 (0.060 to 3.333)
		AKIN_Stage 3_: 0.427 (0.057 to 3.183)
HR_adjusted_ (95% CI)	1.139 (0.508 to 2.552)	AKIN_Stage 1_: 1.119 (0.423 to 2.959)
		AKIN_Stage 2_: 0.287 (0.034 to 2.410)
		AKIN_Stage 3_: 0.171 (0.018 to 1.637)
Wald statistic	0.100	3.629
*P* value	0.752	0.304
Graft survival		
1-year graft survival		
HR (95% CI)	1.124 (0.575 to 2.199)	AKIN_Stage 1_: 1.226 (0.421 to 3.565)
		AKIN_Stage 2_: 0.987 (0.340 to 2.867)
		AKIN_Stage 3_: 1.169 (0.474 to 2.887)
HR_adjusted_ (95% CI)	1.138 (0.480 to 2.696)	AKIN_Stage 1_: 1.232 (0.307 to 4.946)
		AKIN_Stage 2_: 0.688 (0.144 to 3.292)
		AKIN_Stage 3_: 1.632 (0.423 to 6.296)
Wald statistic	0.086	0.701
*P* value	0.769	0.873
1-year death-censored graft survival		
HR (95% CI)	1.286 (0.639 to 2.590)	AKIN_Stage 1_: 1.410 (0.475 to 4.183)
		AKIN_Stage 2_: 1.115 (0.377 to 3.297)
		AKIN_Stage 3_: 1.347 (0.533 to 3.403)
HR_adjusted_ (95% CI)	1.268 (0.505 to 3.185)	AKIN_Stage 1_: 1.271 (0.282 to 5.719)
		AKIN_Stage 2_: 0.787 (0.148 to 4.181)
		AKIN_Stage 3_: 1.814 (0.445 to 7.390)
Wald statistic	0.255	0.738
*P* value	0.613	0.864
3-year graft survival		
HR (95% CI)	1.091 (0.596 to 1.994)	AKIN_Stage 1_: 1.456 (0.556 to 8.816)
		AKIN_Stage 2_: 0.773 (0.270 to 2.208)
		AKIN_Stage 3_: 1.147 (0.521 to 2.524)
HR_adjusted_ (95% CI)	0.609 (0.202 to 1.835)	AKIN_Stage 1_: 1.029 (0.156 to 6.802)
		AKIN_Stage 2_: 0.360 (0.057 to 2.259)
		AKIN_Stage 3_: 0.736 (0.159 to 3.414)
Wald statistic	0.776	1.393
*P* value	0.378	0.707
3-year death-censored graft survival		
HR (95% CI)	1.220 (0.643 to 2.315)	AKIN_Stage 1_: 2.104 (0.702 to 6.309)
		AKIN_Stage 2_: 0.823 (0.284 to 2.384)
		AKIN_Stage 3_: 1.271 (0.567 to 2.852)
HR_adjusted_ (95% CI)	0.549 (0.149 to 2.025)	AKIN_Stage 1_: 1.117 (0.117 to 10.678)
		AKIN_Stage 2_: 0.283 (0.034 to 2.378)
		AKIN_Stage 3_: 0.745 (0.139 to 3.981)
Wald statistic	0.810	1.632
*P* value	0.368	0.652
Graft function		
3-months eGFR		
Standardized *b* coefficient (95% CI)	0.022 (− 4.237 to 5.946)	0.049 (− 1.375 to 2.981)
Adjusted standardized *b* coefficient (95% CI)	− 0.149 (− 11.114 to 0.604)	** − 0.195 (− 5.239 to − 0.431)**
*F* test	2.67	2.82
*P* value	0.078	0.021
1-year eGFR		
Standardized *b* coefficient (95% CI)	− 0.111 (− 9.322 to 0.818)	− 0.088 (− 3.620 to 0.732)
Adjusted standardized *b* coefficient (95% CI)	** − 0.179 (− 13.728 to 0.316)**	** − 0.194 (− 5.966 to − 0.282)**
*F* test	2.12	2.16
*P* value	0.040	0.031
3-year eGFR		
Standardized *b* coefficient (95% CI)	− 0.028 (− 8.359 to 6.437)	− 0.064 (− 3.995 to 2.164)
Adjusted standardized *b* coefficient (95% CI)	0.091 (− 2.381 to 7.418)	0.101 (− 0.856 to 3.054)
*F* test	11.47	11.53
*P* value	< 0.001	< 0.001

*AKI* acute kidney injury, *OR* odds ratio, *HR* hazard ratio, *CI* confidence interval, *eGFR* estimated glomerular filtration rate

Numbers in bold are statistically significant (for those with a *P* < 0.05)

*No patient died in Group AKIN stage 3 after 12 months

Cox proportional hazard models adjusted for multiple clinical and histological variables (Supplementary File 1) showed that there was no significant association between AKI stage and patient survival, death-censored and non-death-censored graft failure (Table 8). Similar results were also observed for 5 years after transplantation (data not shown).

Interestingly, there was weak evidence of an association between 3- and 12-month recipient eGFR and increasing stage of donor AKI (Table 8). The models performed similarly, but the adjusted *R*^2^ values were worse (*R* < 0.30 for all models).

Results on graft survival are illustrated in Fig. 2. Between non-AKI and AKI donors, the outcome of recipients with and without DGF was calculated (Supplementary Table 1). Patient survival was unaffected in all four groups. Overall, death-censored graft survival was lower in the DGF and AKI groups, but there was a significant interaction between both. Transplant patients experiencing DGF and receiving kidneys from donors with AKI exhibited the lowest graft survival (Fig. 2D).

## Discussion

The deceased donor pool is limited and living kidney donation does not suffice to close the gap in organ shortage. For donors with AKI, several aspects have to be taken into account, such as surgical issues, hemodynamic compromise, immunological issues and ischemia reperfusion injury [19]. Unfortunately, donors with AKI are either not considered for donation or kidneys from such donors are often discarded during multi-organ harvest [20]. The aim of this analysis was to assess the impact of donor AKI, as classified by the AKIN criteria, on outcomes in KT of marginal organs. Additionally, a total of 223 post-explantation biopsies of 141 donors with marginal kidneys were analyzed to find possible decisive factors that are relevant for the outcome of the transplantation.

The main findings of our study were:

1. Short-term patient and allograft survival and graft function appear acceptable after transplantation of marginal kidneys with and without AKI to expand the donor pool.
2. The incidence of DGF was significantly higher only in recipients of kidneys from marginal donor kidneys with AKIN stage 3, but not in recipients of donor kidneys with AKIN stage 1 and 2.
3. Donor AKI and recipient DGF in combination were considerably associated with graft loss.
4. Histopathological assessment of donor kidneys was not helpful in predicting outcomes;
5. The rate of cumulative rejections or the level of proteinuria at follow-up was not higher in recipients of marginal donor kidneys with AKI.

Similar to others, we confirmed classical risk factors for AKI, such as diabetes mellitus, BMI and preexisting chronic kidney disease [21–23]. Moreover, the longer duration of ICU stay, and the less common use of steroids and volume expanders in the donor AKI group emphasize the importance of the systemic inflammatory response and intravascular volume depletion respectively as modifiable risk factors for AKI in the ICU setting [24–26]. Donor age was surprisingly lower in the donor AKI group. We suppose that the presumed better organ quality associated with lower age was reassuring but the relative benefit of age was here outweighed by the risk associated with the other above-mentioned factors.

The discrepancy between macroscopic and microscopic findings in the no-AKI group (good morphology, bad histology) implicates that macroscopic assessment of a recovered organ from transplant team is a subjective and probably not accurate parameter of organ quality. Furthermore, it underpins the pitfalls of procurement biopsies in marginal kidneys and corroborates the allocation policy of the European senior program (ESP) of Euro Transplant (ET), where biopsy is not a prerequisite and indeed is not performed in the great majority of the recovered organs. Regarding histopathology, we suppose that the opposite as expected patterns in donor kidneys with AKI are due to selection bias, since only post-explantation biopsies of marginal kidneys were assessed. In that cases, histological findings are unfavorable in general and differences between marginal donor kidneys with and without AKI would not be anticipated. This was also probably the reason why, except for DGF, histological findings failed to predict clinical outcomes, in the multivariate analysis. Perhaps, the statistical analysis did not confirm the significance of these lesions, although their clinical relevance was evident, i.e., that AKI kidneys could be transplanted with satisfactory outcomes. Hence, our results do not support the routinely performance of procurement biopsies in deceased donor kidneys with AKI.

The occurrence of DGF in presence of donor AKI is plausible but the extremely poor outcomes after concurrence of both unfavorable conditions are probably due to the superimposed damage in the transplanted organ before recovery from AKI. The development and severity of AKI are known risk factors for the transition to chronic- or end-stage renal disease [27]. What’s more in the case of transplantation, is the experimental evidence linking acute epithelial tubular cell injury with an augmentation in the immunogenicity of the allograft, although this has not been confirmed in clinical studies [28]. Similar to other studies, our results reveal no disadvantage of transplanting donor kidneys with AKI stage 1 or 2, and call for caution when using AKI stage 3 donor kidneys [9, 29].

The clinical implication of our findings is that patients at high risk of developing DGF should be cautiously selected if kidneys with AKI are offered and in the case of transplantation, careful observation is required during the first three months of follow-up. Such a time frame is of relevance for the allocation policy of ET since patients can be relisted without losing previous accrued waiting-list points in case of graft failure. Insisting on rescuing a graft deemed to get lost could finally result in much longer waiting times for a second transplant. Giving-up the graft and re-initiating dialysis timely is therefore crucial for those patients.

Strengths of the study are the analysis of detailed donor items, concerning the treatment during the ICU stay and the explantation procedure, with data on kidney function from admission to the ICU until recovery, elaborate hemodynamic parameters, concomitant medications and histopathological scoring of the procurement biopsies in a center with experienced nephropathology.

Limitations of our study were the small sample size, the retrospective design, the amount of missing data and the bias toward marginal organs. Lastly, quality of life was not investigated. This is a fundamental item considering the significantly inferior outcomes in graft survival.

In conclusion, our results suggest that transplantation of marginal kidneys with AKI puts recipients in disadvantage only regarding transplanted organ survival and function but not patient survival and may be recommended, always considering an unfavorable risk-to-benefit ratio. Since functioning grafts in the long run outperform dialysis, an individualized approach in the context of a personalized medicine strategy is essential. The non-acceptance of an organ leads in the end to an increase in patients’ mortality due to their longer time on the waiting list [30].

## Supplementary Information

Below is the link to the electronic supplementary material.

Supplementary file1 (DOCX 18 kb)

Supplementary file2 (DOCX 25 kb)
