# Supplementary material for: Short-term outcomes after transplantation of deceased donor kidneys with acute kidney injury: a retrospective analysis of a multicenter cohort of marginal donor kidneys with post-explantation biopsies
Source: Int Urol Nephrol. 2022 Jul 9;55(1):115–27. doi: 10.1007/s11255-022-03277-3 (PMC9807548; doi:10.1007/s11255-022-03277-3)
Supplement: Supplementary file 1 — Supplementary file1 (DOCX 18 kb) [file 11255_2022_3277_MOESM1_ESM.docx]

# Supplementary File 1. Variables of the adjusted models.

Detailed below is a description of the variables that each of the models were adjusted for.

## Primary non function (PNF)

The PNF model was adjusted for

- **Donor factors:** time brain death until cross clamp, lowest creatinine during ICU interval
- **Macro- and histopathological factors:** perfusion quality, g, ci, IFTA
- **Recipient factors:** diabetes, MMB
- **Transplant factors:** cold ischemic time, warm ischemic time

## Delayed Graft Function (DGF)

The DGF model was adjusted for

- **Donor factors:** urine volume last hour before cross clamp per kg, urine protein
- **Macro- and histopathological factors:** organ quality, TMA
- **Recipient factors:** time of dialysis before tx, BMI
- **Transplant factors:** warm ischemic time

## 1-year patient survival

The 1-year patient survival model was adjusted for

- **Donor factors:** age, KDRI, ECD, time ICU until cross clamp
- **Macro- and histopathological factors:**
- **Recipient factors:** age, EPTS, MMDR
- **Transplant factors:** -

**3-years patient survival**

The 3-years patient survival model was adjusted for

- **Donor factors:** age, cardiovascular diseases, KDRI, KDPI, ECD, volume expander
- **Macro- and histopathological factors:** -
- **Recipient factors:** MMDR
- **Transplant factors:** -

## 1-year death-censored graft survival

The 1-year death-censored graft survival model was adjusted for

- **Donor factors:** urine volume last 24 hours before cross clamp per kg, ECD
- **Macro- and histopathological factors:** organ quality, ci, ct, fibrosis, loss of nuclear staining
- **Recipient factors:** HbsAg
- **Transplant factors:** DGF

**3-years death-censored graft survival**

The 3-years death-censored graft survival model was adjusted for

- **Donor factors:** age, sex, BMI, hypertension, HCV, ECD
- **Macro- and histopathological factors:** organ quality, ci, ct, IFTA, fibrosis
- **Recipient factors:** Age
- **Transplant factors:** DGF

## 3-months eGFR

The 3-months eGFR models was adjusted for

- **Donor factors:** age, sex, BMI, diabetes, hypertension, ECD, KDRI, KDPI, time incision until cross clamp
- **Macro- and histopathological factors:** t, ct, IFTA, TMA, detritus
- **Recipient factors:** age, sex, CMV, duration of HD, MMB, EPTS, EPTS Grading
- **Transplant factors:** cold ischemia time

**1-year eGFR**

The 1-year eGFR models was adjusted for

- **Donor factors:** KDRI, KDPI, CPR, time ICU until death, time incision until cross clamp, hypertension, cardiovascular diseases, ECD
- **Macro- and histopathological factors:** t, ct, IFTA
- **Recipient factors:** BMI, MMB
- **Transplant factors:** -

**3-years eGFR**

The 3-years eGFR models was adjusted for

- **Donor factors:** age, time ICU until death, ECD, hematocrit, creatinine at admission
- **Macro- and histopathological factors:** perfusion quality, organ quality, detritus, epithet cell flattening
- **Recipient factors:** MMB, MMDR
- **Transplant factors:** GFR at 12 months, delayed graft function
