# Supplementary material for: Short-term outcomes after transplantation of deceased donor kidneys with acute kidney injury: a retrospective analysis of a multicenter cohort of marginal donor kidneys with post-explantation biopsies
Source: Int Urol Nephrol. 2022 Jul 9;55(1):115–27. doi: 10.1007/s11255-022-03277-3 (PMC9807548; doi:10.1007/s11255-022-03277-3)
Supplement: Supplementary file 2 — Supplementary file2 (DOCX 25 kb) [file 11255_2022_3277_MOESM2_ESM.docx]

Supplementary Table 1. Outcome of recipients from donor kidneys with and without AKI and/or with and without DGF.

|  | Non-AKI, Non-DGF  (n = 63) | Non-AKI, DGF  (n = 54) | AKI, Non-DGF  (n = 25) | AKI, DGF  (n = 55) | P Value |
| --- | --- | --- | --- | --- | --- |
| Patient survival at 1 year - n (%) | 56 (88.9) | 48 (88.39 | 24 (96.0) | 51 (92.7) | 0.665 |
| Patient survival at 3 years - n (%) | 54 (85.7) | 46 (85.2) | 23 (92.0) | 47 (85.5) | 0.851 |
| Patient survival at 5 years - n (%) | 52 (82.5) | 42 (77.8) | 23 (92.0) | 46 (83.6) | 0.482 |
|  |  |  |  |  |  |
| Death censored graft survival at 1 year - n (%) | 56 (100.0) | 41 (85.4) | 22 (91.7) | 44 (86.3) | **0.032** |
| Death censored graft survival at 3 years - n (%) | 51 (94.4) | 37 (80.4) | 20 (87.0) | 39 (83.0) | 0.182 |
| Death censored graft survival at 5 years - n (%) | 45 (91.8) | 30 (76.9) | 20 (87.0) | 38 (82.6) | 0.262 |
|  |  |  |  |  |  |
| Creatinine at 3 months - mmol/l | 169.9 ± 60.1 | 206.8 ± 100.7 | 162.6 ± 52.2 | 195.8 ± 89.7 | **0.026** |
| eGFR at 3 months - ml/min per 1.73 m^2^ | 38.4 ± 16.9 | 34.9 ± 20.2 | 39.3 ± 14.1 | 36.6 ± 20.6 | 0.665 |
| Creatinine at 1 year - mmol/l | 154.8 ± 58.6 | 176.2 ± 64.1 | 167.3 ± 45.9 | 176.3 ± 53.9 | 0.121 |
| eGFR at 1 year - ml/min per 1.73 m^2^ | 43.1 ± 18.3 | 39.1 ± 24.0 | 37.5 ± 14.5 | 36.5 ± 13.3 | 0.236 |
| Creatinine at 3 years - mmol/l | 154.1 ± 58.8 | 185.4 ± 41.2 | 150.2 ± 54.8 | 181.0 ± 72.5 | 0.148 |
| eGFR at 3 years - ml/min per 1.73 m^2^ | 43.9 ± 20.0 | 32.0 ± 7.8 | 45.3 ± 18.0 | 36.2 ± 14.9 | **0.047** |
|  |  |  |  |  |  |
| Proteinuria at 3 months - g/day | 0.47 ± 1.77 | 0.19 ± 0.14 | 0.39 ± 0.34 | 0.22 ± 0.16 | 0.680 |
| Proteinuria at 1 year - g/day | 0.17 ± 0.06 | 0.46 ± 0.94 | 0.34 ± 0.34 | 0.23 ± 0.19 | 0.214 |
|  |  |  |  |  |  |
| Number of rejections | 0.74 ± 1.42 | 0.63 ± 0.79 | 0.56 ± 0.89 | 0.61 ± 0.99 | 0.931 |

Abbreviations: AKI, acute kidney injury; DGF, Delayed Graft Function; g, gram; l, liter; min, minute; ml, milliliter; mmol, millimole; m^2^, square meter.
